# Supplementary material for: Influences of environment, human activity, and climate on the invasion of Ageratina adenophora (Spreng.) in Southwest China
Source: PeerJ. 2023 Mar 9;11:e14902. doi: 10.7717/peerj.14902 (PMC10008309; doi:10.7717/peerj.14902)
Supplement: Supplemental Information 1 [file peerj-11-14902-s001.zip › file 1/Appendix 2 Species lists.docx]

Appendix 2 Species lists

| Index | Species | Genus | Family | Growth Form | Native/Alien |
| --- | --- | --- | --- | --- | --- |
| 1 | *Pinus yunnanensis* | *Pinus* | Pinaceae | Tree | Native plant |
| 2 | *Quercus aliena* | *Quercus* | Fagaceae | Tree | Native plant |
| 3 | *Quercus variabilis* | *Quercus* | Fagaceae | Tree | Native plant |
| 4 | *Lyonia ovalifolia* | *Lyonia* | Ericaceae | Tree | Native plant |
| 5 | *Viburnum cylindricum* | *Viburnum* | Adoxaceae | Tree | Native plant |
| 6 | *Pinus armandi* | *Pinus* | Pinaceae | Tree | Native plant |
| 7 | *Ternstroemia gymnanthera* | *Ternstroemia* | Pentaphylacaceae | Tree | Native plant |
| 8 | *Cunninghamia lanceolata* | *Cunninghamia* | Cupressaceae | Tree | Native plant |
| 9 | *Dodonaea viscosa* | *Dodonaea* | Sapindaceae | Tree | Native plant |
| 10 | *Cyclobalanopsis glauca* | *Cyclobalanopsis* | Fagaceae | Tree | Native plant |
| 11 | *Pyracantha crenulata* | *Pyracantha* | Rosaceae | Tree | Native plant |
| 12 | *Eucalyptus robusta* | *Eucalyptus* | Myrtaceae | Tree | Alien plant |
| 13 | *Sapindus mukorossi* | *Sapindus* | Sapindaceae | Tree | Native plant |
| 14 | *Litsea cubeba* | *Litsea* | Lauraceae | Tree | Native plant |
| 15 | *Lithocarpus glaber* | *Lithocarpus* | Fagaceae | Tree | Native plant |
| 16 | *Keteleeria evelyniana* | *Keteleeria* | Pinaceae | Tree | Native plant |
| 17 | *Pyrus pashia* | *Pyrus* | Rosaceae | Tree | Native plant |
| 18 | *Cyclobalanopsis gambleana* | *Cyclobalanopsis* | Fagaceae | Tree | Native plant |
| 19 | *Toxicodendron succedaneum* | *Toxicodendron* | Anacardiaceae | Shrub | Native plant |
| 20 | *Quercus monimotricha* | *Quercus* | Fagaceae | Shrub | Native plant |
| 21 | *Lyonia ovalifolia* | *Lyonia* | Ericaceae | Shrub | Native plant |
| 22 | *Synotis cappa* | *Synotis* | Asteraceae | Shrub | Native plant |
| 23 | *Elsholtzia rugulosa* | *Elsholtzia* | Lamiaceae | Shrub | Native plant |
| 24 | *Leptodermis pilosa var. pilosa* | *Leptodermis* | Rubiaceae | Shrub | Native plant |
| 25 | *Rubus coreanus* | *Rubus* | Rosaceae | Shrub | Native plant |
| 26 | *Rhus chinensis* | *Rhus* | Anacardiaceae | Shrub | Native plant |
| 27 | *Berchemia yunnanensis* | *Berchemia* | Rhamnaceae | Shrub | Native plant |
| 28 | *Rhododendron racemosum* | *Rhododendron* | Ericaceae | Shrub | Native plant |
| 29 | *Viburnum kansuense* | *Viburnum* | Adoxaceae | Shrub | Native plant |
| 30 | *Barleria cristata* | *Barleria* | Acanthaceae | Shrub | Native plant |
| 31 | *Lespedeza floribunda* | *Lespedeza* | Fabaceae | Shrub | Native plant |
| 32 | *Aster albescens* | *Aster* | Asteraceae | Shrub | Native plant |
| 33 | *Rhododendron siderophyllum* | *Rhododendron* | Ericaceae | Shrub | Native plant |
| 34 | *Myrsine africana* | *Myrsine* | Primulaceae | Shrub | Native plant |
| 35 | *Cotoneaster microphyllus* | *Cotoneaster* | Rosaceae | Shrub | Native plant |
| 36 | *Elaeagnus umbellata* | *Elaeagnus* | Elaeagnaceae | Shrub | Native plant |
| 37 | *Rosa soulieana* | *Rosa* | Rosaceae | Shrub | Native plant |
| 38 | *Callerya congestiflora* | *Callerya* | Fabaceae | Shrub | Native plant |
| 39 | *Hypericum patulum* | *Hypericum* | Hypericaceae | Shrub | Native plant |
| 40 | *Rosa sweginzowii* | *Rosa* | Rosaceae | Shrub | Native plant |
| 41 | *Pyracantha fortuneana* | *Pyracantha* | Rosaceae | Shrub | Native plant |
| 42 | *Prinsepia utilis* | *Prinsepia* | Rosaceae | Shrub | Native plant |
| 43 | *Vaccinium fragile* | *Vaccinium* | Ericaceae | Shrub | Native plant |
| 44 | *Eurya hebeclados* | *Eurya* | Pentaphylacaceae | Shrub | Native plant |
| 45 | *Camellia japonica* | *Camellia* | Theaceae | Shrub | Native plant |
| 46 | *Vernonia esculenta* | *Vernonia* | Asteraceae | Shrub | Native plant |
| 47 | *Urena lobata* | *Urena* | Malvaceae | Shrub | Native plant |
| 48 | *Coriaria nepalensis* | *Coriaria* | Coriariaceae | Shrub | Native plant |
| 49 | *Clematis armandii* | *Clematis* | Ranunculaceae | Shrub | Native plant |
| 50 | *Indigofera amblyantha* | *Indigofera* | Fabaceae | Shrub | Native plant |
| 51 | *Rubus ellipticus var. obcordatus* | *Rubus* | Rosaceae | Shrub | Native plant |
| 52 | *Pyracantha crenulata* | *Pyracantha* | Rosaceae | Shrub | Native plant |
| 53 | *Litsea cubeba* | *Litsea* | Lauraceae | Shrub | Native plant |
| 54 | *Glochidion puberum* | *Glochidion* | Phyllanthaceae | Shrub | Native plant |
| 55 | *Phyllodium elegans* | *Phyllodium* | Fabaceae | Shrub | Native plant |
| 56 | *Reinwardtia indica* | *Reinwardtia* | Linaceae | Shrub | Native plant |
| 57 | *Maesa japonica* | *Maesa* | Primulaceae | Shrub | Native plant |
| 58 | *Ficus tikoua* | *Ficus* | Moraceae | Shrub | Native plant |
| 59 | *Quercus variabilis* | *Quercus* | Fagaceae | Shrub | Native plant |
| 60 | *Dodonaea viscosa* | *Dodonaea* | Sapindaceae | Shrub | Native plant |
| 61 | *Carex baccans* | *Carex* | Cyperaceae | Herb | Native plant |
| 62 | *Arthraxon lanceolatus* | *Arthraxon* | Poaceae | Herb | Native plant |
| 63 | *Polygonum capitatum* | *Polygonum* | Polygonaceae | Herb | Native plant |
| 64 | *Deyeuxia pyramidalis* | *Deyeuxia* | Poaceae | Herb | Native plant |
| 65 | *Clematis montana* | *Clematis* | Ranunculaceae | Herb | Native plant |
| 66 | *Bidens pilosa* | *Bidens* | Asteraceae | Herb | Alien plant |
| 67 | *Pteridium aquilinum var. latiusculum* | *Pteridium* | Dennstaedtiaceae | Herb | Native plant |
| 68 | *Saccharum arundinaceum* | *Saccharum* | Poaceae | Herb | Native plant |
| 69 | *Notoseris psilolepis* | *Notoseris* | Asteraceae | Herb | Native plant |
| 70 | *Viola delavayi* | *Viola* | Violaceae | Herb | Native plant |
| 71 | *Ageratina adenophora* | *Ageratina* | Asteraceae | Herb | Invasive alien plant |
| 72 | *Rubia edgeworthii* | *Rubia* | Rubiaceae | Herb | Native plant |
| 73 | *Senecio scandens* | *Senecio* | Asteraceae | Herb | Native plant |
| 74 | *Leontopodium artemisiifolium* | *Leontopodium* | Asteraceae | Herb | Native plant |
| 75 | *Heteropogon contortus* | *Heteropogon* | Poaceae | Herb | Native plant |
| 76 | *Anaphalis busua* | *Anaphalis* | Asteraceae | Herb | Native plant |
| 77 | *Artemisia sacrorum* | *Artemisia* | Asteraceae | Herb | Native plant |
| 78 | *Ainsliaea yunnanensis* | *Ainsliaea* | Asteraceae | Herb | Native plant |
| 79 | *Cymbidium goeringii* | *Cymbidium* | Orchidaceae | Herb | Native plant |
| 80 | *Carex pergracilis* | *Carex* | Cyperaceae | Herb | Native plant |
| 81 | *Onychium japonicum* | *Onychium* | Pteridaceae | Herb | Native plant |
| 82 | *Fragaria moupenensis* | *Fragaria* | Rosaceae | Herb | Native plant |
| 83 | *Pogonatherum crinitum* | *Pogonatherum* | Poaceae | Herb | Native plant |
| 84 | *Galium paradoxum* | *Galium* | Rubiaceae | Herb | Native plant |
| 85 | *Clinopodium megalanthum* | *Clinopodium* | Lamiaceae | Herb | Native plant |
| 86 | *Agrimonia pilosa* | *Agrimonia* | Rosaceae | Herb | Native plant |
| 87 | *Eremopogon delavayi* | *Eremopogon* | Poaceae | Herb | Native plant |
| 88 | *Oxalis corniculata* | *Oxalis* | Oxalidaceae | Herb | Native plant |
| 89 | *Swertia bimaculata* | *Swertia* | Gentianaceae | Herb | Native plant |
| 90 | *Ophiopogon bodinieri* | *Ophiopogon* | Asparagaceae | Herb | Native plant |
| 91 | *Dryopteris rosthornii* | *Dryopteris* | Dryopteridaceae | Herb | Native plant |
| 92 | *Pteris wallichiana* | *Pteris* | Pteridaceae | Herb | Native plant |
| 93 | *Capillipedium parviflorum* | *Capillipedium* | Poaceae | Herb | Native plant |
| 94 | *Cyperus rotundus* | *Cyperus* | Cyperaceae | Herb | Native plant |
| 95 | *Crassocephalum crepidioides* | *Crassocephalum* | Asteraceae | Herb | Alien plant |
| 96 | *Stellera chamaejasme* | *Stellera* | Thymelaeaceae | Herb | Native plant |
| 97 | *Laggera pterodonta* | *Laggera* | Asteraceae | Herb | Native plant |
| 98 | *Desmodium microphyllum* | *Desmodium* | Fabaceae | Herb | Native plant |
| 99 | *Conyza japonica* | *Conyza* | Asteraceae | Herb | Native plant |
| 100 | *Galinsoga parviflora* | *Galinsoga* | Asteraceae | Herb | Alien plant |
| 101 | *Ainsliaea fragrans* | *Ainsliaea* | Asteraceae | Herb | Native plant |
| 102 | *Ixeris chinensis* | *Ixeris* | Asteraceae | Herb | Native plant |
| 103 | *Gerbera piloselloides* | *Gerbera* | Asteraceae | Herb | Native plant |
| 104 | *Ajuga ciliata* | *Ajuga* | Lamiaceae | Herb | Native plant |
| 105 | *Pseudostellaria heterophylla* | *Pseudostellaria* | Caryophyllaceae | Herb | Native plant |
| 106 | *Phlomis umbrosa* | *Phlomis* | Lamiaceae | Herb | Native plant |
| 107 | *Carpesium abrotanoides* | *Carpesium* | Asteraceae | Herb | Native plant |
| 108 | *Paederia scandens var. scandens* | *Paederia* | Rubiaceae | Herb | Native plant |
| 109 | *Parthenocissus semicordata* | *Parthenocissus* | Vitaceae | Herb | Native plant |
| 110 | *Rubus parvifolius* | *Rubus* | Rosaceae | Herb | Native plant |
| 111 | *Origanum vulgare* | *Origanum* | Lamiaceae | Herb | Native plant |
| 112 | *Rabdosia parvifolia* | *Rabdosia* | Lamiaceae | Herb | Native plant |
| 113 | *Polygala tenuifolia* | *Polygala* | Polygalaceae | Herb | Native plant |
| 114 | *Cynoglossum zeylanicum* | *Cynoglossum* | Boraginaceae | Herb | Native plant |
