# Supplementary figures and images for: Influences of environment, human activity, and climate on the invasion of Ageratina adenophora (Spreng.) in Southwest China
Source: PeerJ. 2023 Mar 9;11:e14902. doi: 10.7717/peerj.14902 (PMC10008309; doi:10.7717/peerj.14902)

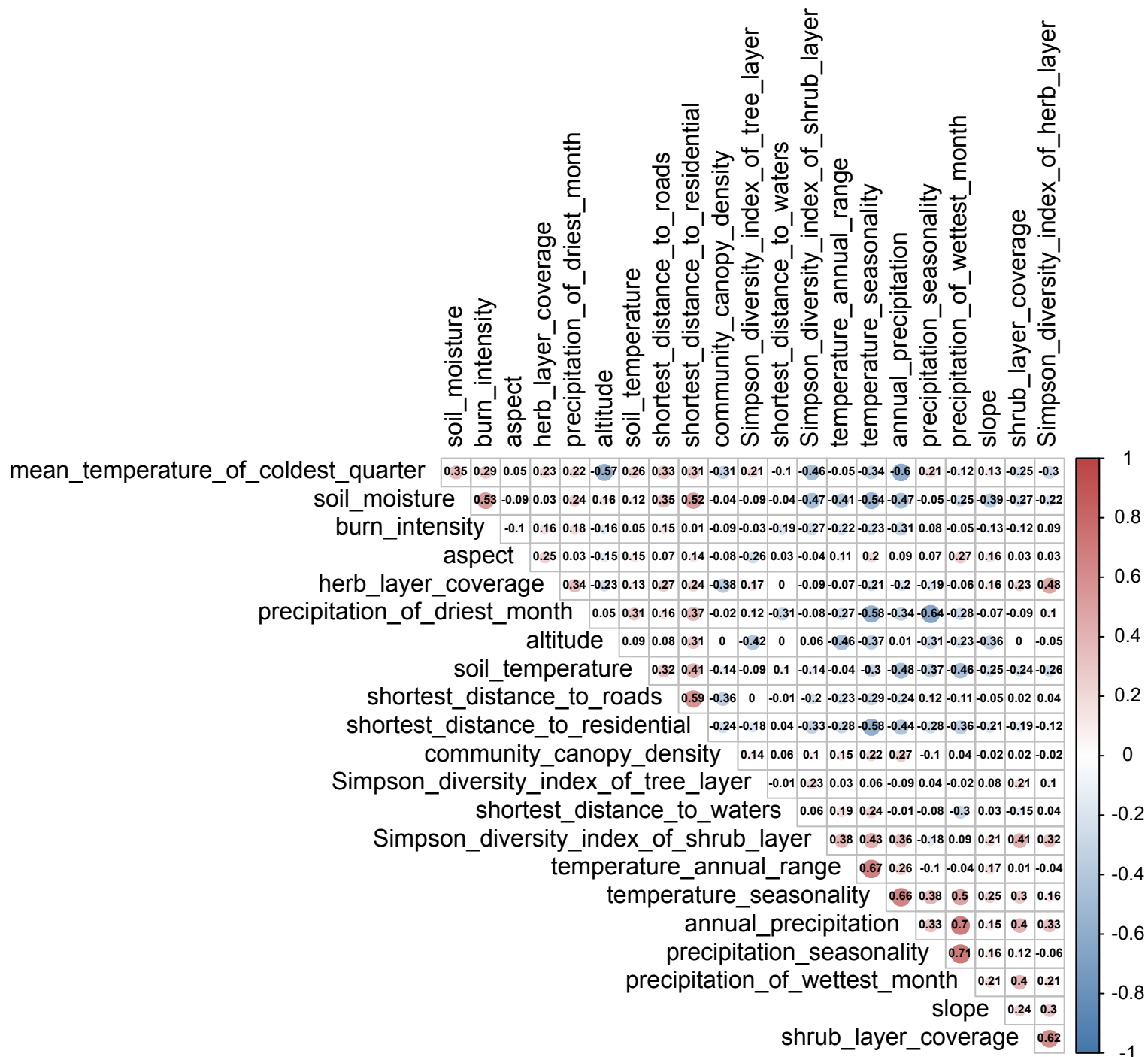

Supplement: Supplemental Information 1 [file peerj-11-14902-s001.zip › file 1/Appendix 1.pdf]
